# Liposarcome paratesticulaire métastatique

**DOI:** 10.11604/pamj.2017.27.101.12687

**Published:** 2017-06-08

**Authors:** Otheman Fahsi, Adil Kallat, Hicham Ouazize, Hamza Dergamoun, Hachem El Sayegh, Ali Iken, Lounis Benslimane, Yassine Nouini

**Affiliations:** 1 Service d’Urologie A, Hôpital Ibn Sina, CHU Rabat, Maroc

**Keywords:** Liposarcome, paratesticulaire, orchidéctomie, Liposarcoma, paratesticular, orchidectomy

## Abstract

Nous rapportons le cas dramatique d'un liposarcome à cellules rondes paratesticulaire d'emblée métastatique chez un jeune homme de 18 ans. Il s'agit d'une tumeur rare qui se développe à partir du tissu graisseux entourant le testicule et le cordon spermatique. Les signes cliniques et radiologiques sont peu spécifiques, le diagnostic n'est en général fait que sur la pièce opératoire. Le traitement consiste en une orchidéctomie radicale par voie inguinale, parfois élargie aux structures adjacentes. La radiothérapie adjuvante pourrait avoir une place en cas de masse localement avancée ou en cas de résection incomplète. Malgré une évolutivité lente, une surveillance prolongée s'impose du fait du risque élevé de récidive tardive.

## Abstract (en)

We report the dramatic case of a 18-year old patient with immediately metastatic round cells paratesticular liposarcoma. It is a rare tumor that develops in the fatty tissue surrounding the testicle and the spermatic cord. Clinical and radiological signs are nonspecific and diagnosis is usually based on surgical specimen examination. The treatment involves radical inguinal orchiectomy, sometimes extended to adjacent structures. Adjuvant radiation therapy could be used in the case of locally advanced mass or incomplete resection. Despite its slow progression, prolonged monitoring is required due to the high risk of late recurrence.

## Introduction

Le liposarcome para-testiculaire se développe à partir du tissu graisseux entourant de cordon spermatique et recouvre le testicule et l'épididyme. C'est une entité pathologique extrêmement rare [1]. Environ 200 cas ont été rapportés dans la littérature, mais leur incidence a augmenté lentement. Les rayonnements, le déficit immunitaire, certains médicaments, certains facteurs héréditaires et certains virus pourraient jouer un rôle dans leur pathogenèse [2]. Il n'y a pas de prise en charge standardisée en raison de la rareté des cas dans la littérature.

## Patient et observation

**Figure 1:**
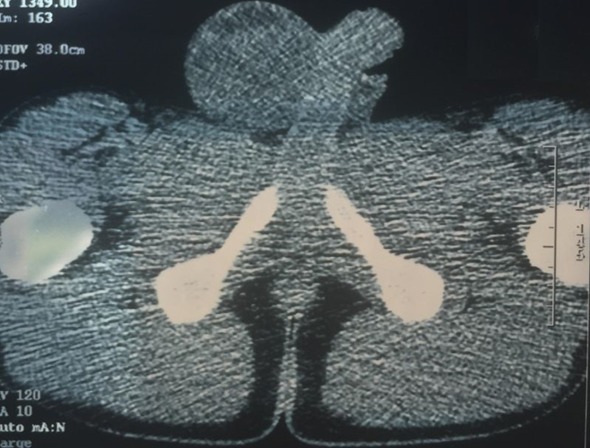
Image tomodensitométrique inguinoscrotal montrant une grande masse s’étendant vers le canal inguinal droit

**Figure 2:**
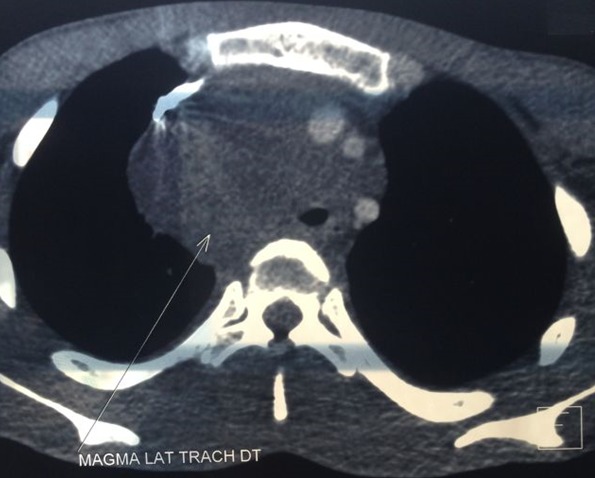
Image tomodensitométrique thoracique montrant un magma d’adénopathies latéro-trachéal refoulant et laminant la veine cave supérieure et la trachée

**Figure 3:**
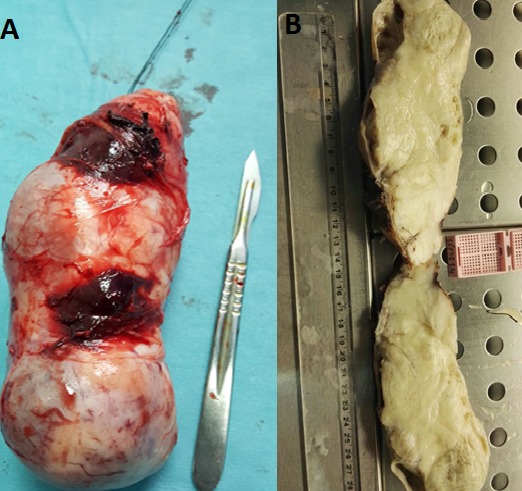
(A) pièce opératoire d’orchidéctomie par voie haute emportant toute la masse inguino-scrotale; (B) la coupe présence d’une tumeur d’aspect blanchâtre fasciculée avec des remaniements myxoides avec envahissement totale du testicule et de l’épididyme

**Figure 4:**
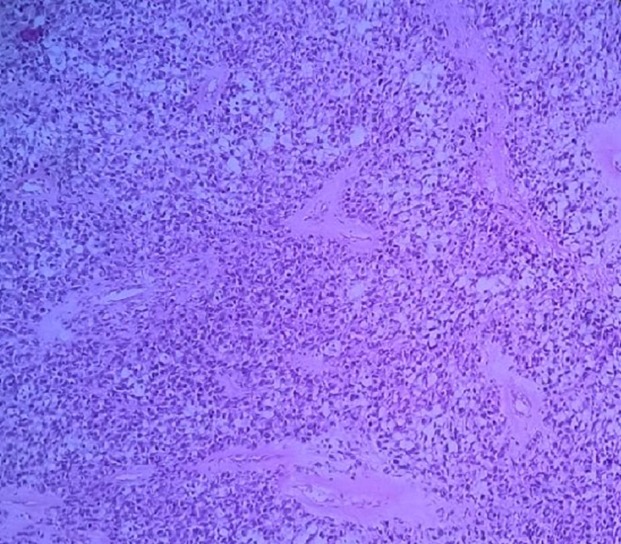
Prolifération tumorale, faite de cellules rondes à grands noyaux discrètement anisocaryotiques et hyperchromatiques sur un fond myxoïde

Jeune homme de 18 ans sans antécédents pathologiques notables, admis pour prise en charge d'une masse inguino-scrotale droite indolore apparue il y a 3 mois, associée à des lombalgies du même côté, toux avec crachats verdâtres et une altération de l'état général. L'examen général trouve un patient en mauvais état général, polypneique à 25 cycles par minute râles ronflants avec des conjonctives décolorées. L'examen clinique retrouve une volumineuse tuméfaction de l'hémiscrotum droit, indolore, ferme et irrégulière. Le testicule l'épididyme et le cordon spermatique ne sont pas individualisables au sein de cette masse non transilluminable. Cette masse se continue dans le canal inguinal avec une autre masse abdomino-pelvienne remontant jusqu'au flanc droit. Le bilan biologique retrouve une insuffisance rénale sévère clairance de créatinine à 26ml/min, une bicytopénie: hémoglobine 4.9g/dl, taux de plaquette 52.10^3^/μl, Les marqueurs tumoraux (alpha-foeto -proteine, HCG, LDH) étaient normaux. Le scanner thoraco-abdomino-pelvien (Figure 1) a objectivé une volumineuse masse abdomino-pelvienne et scrotale droite de densité tissulaire, de contours polylobés, décollant l'aorte abdominale et entrainant une hydronéphrose droite. A l'étage thoracique (Figure 2): magma d'adénopathies latéro-trachéal refoulant et laminant la veine cave supérieure et la trachée. Après mise en condition une orchidectomie première a visée diagnostique a été difficilement réalisée à cause de la taille importante de la tumeur. L'examen anatomo-pathologique macroscopique (Figure 3) montrait une volumineuse tumeur (17 x 6 x 5 cm), le cordon spermatique mesure (2 x1.5 cm). A la coupe présence d'une tumeur d'aspect blanchâtre fasciculée avec des remaniements myxoides avec envahissement totale du testicule et de l'épididyme. L'examen microscopique (Figure 4) montrait une prolifération tumorale, faite de cellules rondes à grands noyaux discrètement anisocaryotiques et hyperchromatiques. Cette prolifération évolue sur un fond myxoïde, renfermant de nombreux capillaires branchés à paroi fine. L'index mitotique est estimé à 14 mitoses/1HPF, avec présence des foyers de nécrose estimé à 30%. Cet aspect évoque un liposarcome à cellules rondes de haut grade de malignité. L'évolution a été marquée par l'aggravation rapide de la détresse respiratoire, l'aggravation de la bicytopénie malgré les transfusions avec la survenue d'épistaxis foudroyante. Le patient est décédé 5 jours après l'orchidectomie.

## Discussion

Le liposarcome représente 20% des tumeurs paratesticulaires de l'adulte et occupe la 3^ème^ place après le léiomyosarcome (32%) et le rhabdomyosarcome (24%) [3]. La région paratesticulaire représente le site le plus fréquent des sarcomes urologiques de l'adulte. La plupart des sarcomes n'ont pas été associés à des facteurs de risque, mais certaines prédispositions environnementales et génétiques ont été suggérées chez une minorité des patients [2]. Le liposarcome de l'albuginé, la vaginal, le cordon spermatique, l'épididyme, la peau scrotale et les testicules sont très rares [1]. Le liposarcome paratesticulaire intéresse le sujet âgé entre 50 et 60 ans [3] avec une tranche d'âge entre 16 à 85 ans. Il siège plus volontiers à droite comme le cas de notre patient. Cliniquement, la lésion évolue le plus souvent à bas bruit pendant plusieurs mois voire des années. Elle se manifeste par une douleur, pesanteur ou un tiraillement dans 10-15% des cas. Elle est ferme, irrégulière, indolore, développée à proximité ou à distance du testicule pouvant être soit intrascrotale ou au niveau de l'aine ou englober les deux, Elle peut prêter confusion avec une hernie inguinale ou une hydrocèle, la taille variable allant de quelques centimètres a plus de 30 cm et peut atteindre des proportions gigantesques jusqu'à 13,5 kg. Il n'y a pas de marqueurs tumoraux pouvant aider au diagnostic. Sur le plan radiologique il n'existe pas de signes radiologiques caractéristiques. La tomodensitométrie ne semble pas supérieure à l'échographie dans l'exploration locale. L'Échographie inguino-scrotale met en évidence typiquement des lésions solides, hyperéchogènes et hétérogènes mais celles-ci peuvent se présenter comme des nodules indurés de petite taille au sein d'un tissu adipeux de consistance proche de la normale, ne permettant pas ainsi la distinction entre les lésions bénignes et malignes. La tomodensitométrie apporte la preuve de la localisation, de l'étendue, et de la relation de la masse intrascrotale avec le testicule, l'épididyme, et le cordon spermatique. Les liposarcomes apparaissent comme des masses graisseuses, comme ils peuvent être hypodenses par rapport à la graisse sous cutanée [4]. Les liposarcomes ont tendance à apparaître bien circonscrits et lobulés sur les IRM [5]. La prise de contraste dépend du niveau de différenciation. Peu de prise de contraste correspond à des liposarcomes bien différenciés alors qu'une prise de contraste est plus importante révèlera des sous-types plus agressifs tels les liposarcomes à cellules rondes, pléïomorphes et dédifférenciés. Les liposarcomes myxoïdes, un sous-type intermédiaire, montrent une hétérogénéité dans la prise de contraste [5]. Les autres aspects caractéristiques des liposarcomes sont d'épaisses cloisons fibreuses, une forme nodulaire et une prise de contraste sur les séquences où le tissu adipeux est supprimé. Des foyers d'hémorragie et de nécrose peuvent également être identifiés. FDG-PET scan peut être utile dans les cas récurrents mais son utilisation en routine n'est pas indiquée.

Sur le plan histologique la nouvelle classification de l'OMS [6] fusionne le liposarcome myxoïde et le liposarcome à cellules rondes, puisque ce dernier est simplement une variante de haut grade du premier, et qu'il est commun de voir une transition entre l'un et l'autre dans la même tumeur. Le liposarcome myxoïde survient classiquement dans les tissus mous profonds des extrémités. Il est généralement accepté que la localisation rétropéritonéale est exceptionnellement rare, et représente plus probablement une métastase ou une forme très myxoïde de liposarcome bien différencié. D'un point de vue morphologique, le liposarcome myxoïde est constitué de cellules ovoïdes d'assez petite taille disposées dans une abondante matrice myxoïde, parcourue d'un réseau capillaire sophistiqué rappelant parfois les mailles d'une clôture. Il est généralement facile de repérer des lipoblastes uni- ou multivacuolaires. Il est également possible de retrouver des foyers de tissu adipeux plus mature dans un liposarcome myxoïde, et ceci ne doit pas empêcher de poser le diagnostic. Le liposarcome myxoïde possède un risque de métastase variant de 20 % à 40 % et qui croît avec la proportion de morphologie « à cellules rondes ». Les métastases peuvent survenir à des sites inhabituels, comme les os, le rétropéritoine ou d'autres sites anatomiques dans les tissus mous des extrémités ou du tronc. Contrairement au liposarcome bien différencié, le risque de métastase n'est jamais négligeable. En immunohistochimie, un marqueur réputé sensible et spécifique a récemment été décrit (NY-ESO-1) [7], mais son utilisation est peu répandue et sa fiabilité reste à démontrer. D'un point de vue génétique, le liposarcome myxoïde est associé dans 95 % des cas à une translocation t(12;16) qui entraîne une juxtaposition FUS-DDIT3 (anciennement FUS-CHOP). Dans un petit nombre de cas, une translocation t(12;22) entraîne la juxtaposition EWSR1-DDIT3. Plusieurs points de bris existent pour chacune de ces translocations, mais ils ne semblent pas avoir de répercussion clinique importante. Les transcrits de fusion peuvent être détectés par rt-PCR et les réarrangements chromosomiques mis en évidence par FISH [8].

L'exérèse chirurgicale avec orchidectomie par voie inguinale est la pierre angulaire du traitement à visée curative [9]. L'hémiscrotectomie peut être justifiée en cas de tumeur extra capsulaire avec envahissement des structures adjacentes. L'objectif carcinologique est la résection en monobloc de la tumeur en marge saine microscopique (R0). La pseudo-capsule qui entoure le liposarcome est constituée d'un front de cellules tumorales densifié qui ne constitue pas un plan de clivage pertinent. La qualité d'exérèse est le facteur pronostique le plus significatif, Le caractère R2 prédit indépendamment le risque de mortalité spécifique. Il n'y a pas de consensus clair concernant l'intérêt du curage ganglionnaire rétropéritonéal, il doit être réservé aux patients chez qui des adénopathies ont été identifiées Le rôle de la radiothérapie est encore incertain. Alors que Coleman [4] (47 patients) a rapporté que la radiothérapie adjuvante ne diminue pas significativement le taux de récidives locales et n'améliorait pas la survie globale, d'autres auteurs ont noté un contrôle plus durable après la chirurgie et la radiothérapie combinée. Selon ces auteurs, un traitement combiné est une modalité qui devrait être envisagée, surtout en cas de tumeurs de haut grade, l'invasion lymphatique, marge de résection envahie, ou en cas de rechute [10]. La zone irradiée doit comprendre la partie proximale du scrotum, et le trajet du canal inguinal, ainsi que les tissus avoisinants et les ganglions pelviens ipsilatéraux. La dose d'irradiation doit être de 60 à 65Gy, durant six semaines. Le rôle de la chimiothérapie dans le traitement des liposarcomes est controversé, elle peut être proposée au cas par cas. La chimiothérapie adjuvante a été signalée dans 2 cas, mais le suivi a été court. La chimiothérapie à la doxorubicine a été utilisée de temps en temps. La récidive locale est le problème principal des sarcomes paratesticulaires. Cette récidive survient dans 30 à 50 % des cas [4]. Elle peut survenir après des délais pouvant dépasser cinq ans. Une surveillance sur plusieurs années est donc requise dans tous les cas.

## Conclusion

Le liposarcome para-testiculaire est une tumeur rare, son diagnostic pré-opératoire reste difficile malgré l'apport des moyens d'imagerie récente. Et de ce fait e diagnostic à envisager en priorité devant toute masse solide palpable au niveau paratesticulaire ou du canal inguinal, adipeuse ou non, est celui de liposarcome. Une orchidectomie totale est indiquée en général et une surveillance est toujours requise étant donné le risque de récidive. La place d'un traitement complémentaire, surtout de la radiothérapie, reste à codifier.

## Conflits d’intérêts

Les auteurs ne déclarent aucun conflit d'intérêts.
